# Associations of insulin resistance-related metabolic indices with albuminuria in primary hypertension without diabetes: a retrospective cross-sectional study

**DOI:** 10.3389/fmed.2026.1959589

**Published:** 2026-10-07

**Authors:** Yan Zhuo, Pengjie Gao, Suining Xu, Suhang Shang, Yiran Yan, Kamila Kamili, Xiyang Li, Danni Wang, Jianfeng Han, Gang Tian

**Affiliations:** 1 Department of Cardiology, The First Affiliated Hospital of Xi'an Jiaotong University, Xi’an, China; 2 Department of Neurology, The First Affiliated Hospital of Xi'an Jiaotong University, Xi’an, China

**Keywords:** albuminuria, insulin resistance, mean arterial pressure, metabolic score for insulin resistance, primary hypertension, triglyceride-glucose body mass index, triglyceride-glucose index

## Abstract

**Background:**

Insulin resistance-related metabolic indices have been associated with albuminuria in general populations and in patients with diabetes, but evidence in patients with primary hypertension without diabetes remains limited. Whether mean arterial pressure (MAP) statistically accounts for part of these associations is unclear.

**Methods:**

We retrospectively analysed cross-sectional data from 509 hospitalised cardiology patients with primary hypertension without diabetes at a single tertiary centre who underwent urinary albumin-to-creatinine ratio (UACR) testing. Albuminuria was defined as a UACR ≥30 mg/g. Associations of the triglyceride-glucose body mass index (TyG-BMI), metabolic score for insulin resistance (METS-IR), and triglyceride-glucose (TyG) index with albuminuria and log_10_-transformed UACR were examined using multivariable logistic and linear regression. Restricted cubic splines (RCS) assessed the shape of the associations. Supplementary logistic models additionally adjusted for MAP, and exploratory indirect-effect analyses assessed statistical indirect components involving MAP.

**Results:**

Among the 509 participants, 99 (19.4%) had albuminuria. After multivariable adjustment, the odds ratios (ORs) for albuminuria per 1-SD increment in TyG-BMI, METS-IR, and the TyG index were 1.610 [95% confidence interval (CI), 1.262–2.055], 1.557 (95% CI, 1.215–1.995), and 1.436 (95% CI, 1.128–1.829), respectively. Corresponding Q4-vs.-Q1 ORs were 2.464 (95% CI, 1.228–4.943), 2.665 (95% CI, 1.268–5.600), and 2.137 (95% CI, 1.092–4.181), with overall categorical *P*-values of 0.018, 0.059, and 0.099, respectively. RCS analyses showed significant overall associations for all three indices, with no statistical evidence of departure from linearity. All three indices were positively associated with log_10_-transformed UACR. After additional MAP adjustment, the continuous associations with albuminuria were attenuated but remained statistically significant for all three indices, whereas the overall tests across quartiles were not statistically significant for any of the three indices. Exploratory analyses suggested statistical indirect components involving MAP for TyG-BMI and METS-IR, but not for the TyG index.

**Conclusions:**

Higher TyG-BMI, METS-IR, and TyG index values were associated with albuminuria and higher UACR in patients with primary hypertension without diabetes. Exploratory analyses suggested statistical indirect components involving MAP for TyG-BMI and METS-IR, but did not establish temporal ordering or causal mediation.

## 1 Introduction

Albuminuria is an important marker of kidney damage and is associated with vascular dysfunction and adverse cardiovascular and kidney outcomes in individuals with hypertension or diabetes (1–4). Previous studies have linked insulin resistance (IR) and related metabolic abnormalities to albuminuria, with much of the available evidence derived from populations with diabetes (5–8). However, these associations remain insufficiently characterised in patients with primary hypertension without diabetes. In this population, albuminuria may indicate differences in hypertension-mediated organ involvement and cardiorenal burden that are not fully captured by blood pressure levels alone (1, 3, 4). Moreover, metabolic abnormalities remain common even in the absence of overt diabetes and may represent an additional dimension relevant to albuminuria (9). Because albuminuria in diabetes occurs within a distinct glycaemic and renal context, findings from diabetic populations may not be directly generalisable to populations with primary hypertension without diabetes (7). Clarifying the associations between IR-related metabolic profiles and albuminuria may therefore provide further insight into the metabolic characteristics associated with albuminuria in this clinically distinct population.

The hyperinsulinaemic–euglycaemic clamp is considered the reference standard for assessing insulin sensitivity but is costly, time-consuming, and impractical in routine clinical practice (10, 11). Non-insulin-based surrogate metabolic indices related to IR have therefore been increasingly used in clinical and epidemiological studies (10, 12). The triglyceride-glucose (TyG) index, derived from fasting plasma glucose (FPG) and triglyceride (TG) levels, is among the most widely studied of these indices and has been positively associated with albuminuria in general-population studies (11–14). Triglyceride-glucose body mass index (TyG-BMI) incorporates body mass index (BMI) into the TyG index, whereas the metabolic score for insulin resistance (METS-IR) integrates BMI, FPG, TG, and high-density lipoprotein cholesterol (HDL-C). Both indices therefore incorporate adiposity-related information in addition to glycaemic and lipid components (15, 16). Population-based evidence has linked METS-IR to elevated urinary albumin-to-creatinine ratio (UACR) or albuminuria (13), while evidence linking TyG-BMI to albuminuria has emerged mainly from populations with diabetes (6, 17, 18). Despite these findings, concurrent evaluation of these IR-related indices in patients with primary hypertension without diabetes remains limited, leaving it uncertain whether similar associations are observed across indices with differing component profiles.

Higher blood pressure is closely associated with albuminuria (1, 3, 19), and IR-related metabolic abnormalities have also been linked to blood pressure elevation (20–23). These relationships provide a plausible basis for considering a hypothesised pathway in which mean arterial pressure (MAP), as a summary measure of average systemic arterial pressure (24), may represent an intermediate haemodynamic component in the association between IR-related metabolic abnormalities and albuminuria. Accordingly, this study aimed to concurrently assess the associations of TyG-BMI, METS-IR, and the TyG index with albuminuria in patients with primary hypertension without diabetes. We hypothesised that higher values of all three indices would be associated with greater odds of albuminuria. We further performed exploratory analyses to assess whether these associations included a statistical indirect component involving MAP.

## 2 Materials and methods

### 2.1 Study design and participants

We retrospectively identified consecutive patients with hypertension who were admitted to the Department of Cardiology at the First Affiliated Hospital of Xi'an Jiaotong University and underwent UACR testing between January 2016 and July 2025. UACR testing was routinely recommended as part of inpatient evaluation for patients with hypertension in our department, primarily to assess renal involvement and hypertension-mediated organ damage, although testing was not completed in every patient. For patients with repeated UACR measurements, the earliest eligible assessment accompanied by complete concurrent glycaemic, lipid, and other required laboratory data was designated the index assessment. Primary and secondary hypertension were classified according to the discharge diagnoses recorded for the hospitalisation during which the index assessment was performed, and patients with secondary hypertension were excluded. Other exclusion criteria were age <18 years, diabetes mellitus, estimated glomerular filtration rate (eGFR) < 60 mL/min/1.73 m^2^, severe comorbidities, acute conditions affecting UACR, and missing data for one or more components required to calculate the IR-related indices, including BMI, FPG, TG, or HDL-C. Diabetes was identified based on a documented diagnosis, use of glucose-lowering medication prescribed for diabetes, or laboratory values in the diabetic range, including FPG ≥7.0 mmol/L or glycated haemoglobin A1c (HbA1c) ≥ 6.5% (25). Sodium-glucose cotransporter 2 inhibitor (SGLT2i) use alone was not considered evidence of diabetes because these agents may be prescribed for other indications (26). Severe comorbidities included advanced malignancy, severe hepatic dysfunction (aminotransferase concentrations >3 times the upper limit of normal), and acute heart failure. Acute conditions included acute infection, such as urinary tract infection, and acute kidney injury. No *a priori* sample size calculation was performed; all consecutive screened patients who met the eligibility criteria during the study period were included. This study was approved by the Ethics Committee of the First Affiliated Hospital of Xi'an Jiaotong University (approval No. 2023-KY-0327), and the requirement for written informed consent was waived because of the retrospective nature of the study. Figure 1 summarises participant selection.

**Figure 1:**
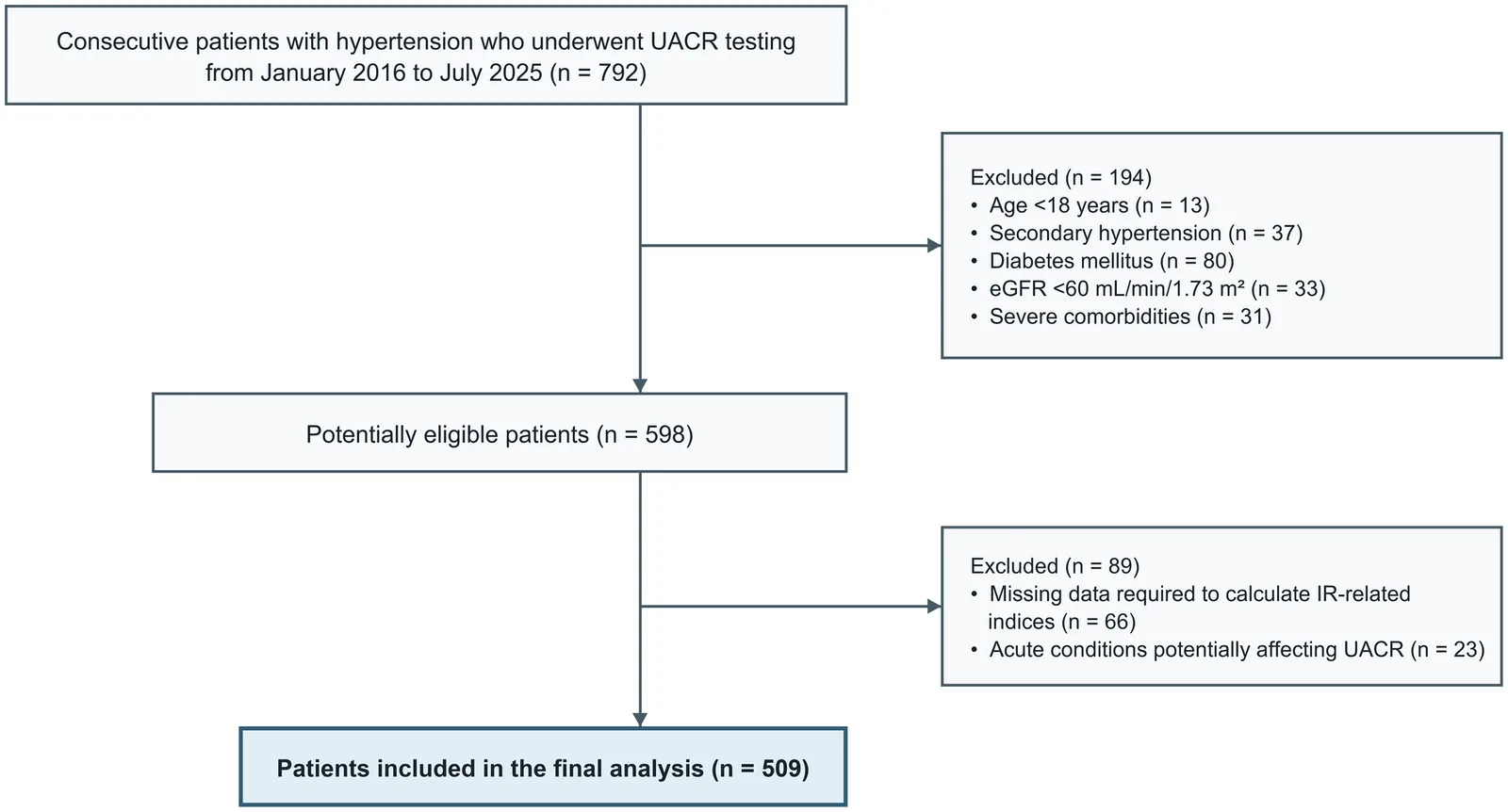
Flow diagram of participant selection. Consecutive patients with hypertension who underwent UACR testing between January 2016 and July 2025 were screened. After applying the exclusion criteria, 509 patients were included in the final analysis. eGFR, estimated glomerular filtration rate; IR, insulin resistance; UACR, urinary albumin-to-creatinine ratio.

### 2.2 Clinical and laboratory data

Medical records were reviewed to collect age, sex, current smoking, current alcohol use, duration of hypertension, history of coronary heart disease (CHD), and medication use. Current smoking and current alcohol use were defined according to the status documented in the medical records at the index admission. Duration of hypertension was based on the patient-reported duration documented in the medical records, and CHD was identified based on a documented clinical diagnosis. Medication use at the index assessment included angiotensin-converting enzyme inhibitor or angiotensin receptor blocker (ACEI/ARB), *β*-blocker, calcium channel blocker (CCB), diuretic, statin, and SGLT2i use. Height, weight, systolic blood pressure (SBP), and diastolic blood pressure (DBP) recorded at the index assessment were extracted from the medical records. Blood pressure was measured using automated devices as part of routine clinical care rather than according to a study-specific standardised protocol. The following equations were used to calculate BMI and MAP:

$$\mathrm{BMI}=\frac{\mathrm{Weight}\;(\mathrm{kg})}{\mathrm{Height}\;(m{)}^{2}}$$

$$\mathrm{MAP}=\frac{\mathrm{SBP}+2\times \mathrm{DBP}}{3}$$

Laboratory variables, including FPG, haemoglobin (Hb), HbA1c, serum creatinine (Scr), serum uric acid (UA), TG, total cholesterol (TC), low-density lipoprotein cholesterol (LDL-C), and HDL-C, were obtained from the hospital laboratory records. Biochemical measurements were based on fasting blood samples collected during the index encounter. eGFR was calculated using the 2021 Chronic Kidney Disease Epidemiology Collaboration creatinine equation (27). A first-morning midstream urine sample collected during the index assessment was used for UACR measurement. Urinary albumin was measured by immunoturbidimetry and urinary creatinine by an enzymatic method. UACR was calculated as the ratio of urinary albumin to urinary creatinine and expressed in mg/g.

### 2.3 IR-related metabolic indices

Three surrogate metabolic indices related to IR, including the TyG index, TyG-BMI, and METS-IR, were calculated according to their original published equations (11, 15, 16). The indices were calculated using the following formulas:

$$\mathrm{TyG}\;\mathrm{index}=\ln\left[\frac{\mathrm{Fasting}\;\mathrm{TG}\;(\mathrm{mg}/\mathrm{dL})\;\times \;\mathrm{FPG}\;(\mathrm{mg}/\mathrm{dL})}{2}\right]$$

TyG-BMI=TyGindex×BMI

$$\mathrm{METS}\text{-}\mathrm{IR}=\frac{\ln\;[2\times \mathrm{FPG}\;(\mathrm{mg}/\mathrm{dL})+\mathrm{Fasting}\;\mathrm{TG}\;(\mathrm{mg}/\mathrm{dL})]\times \mathrm{BMI}\;(\mathrm{kg}/m^{2})}{\ln\;[\mathrm{HDL}\text{-}C\;(\mathrm{mg}/\mathrm{dL})]}$$

### 2.4 Outcome definition

The primary outcome was albuminuria at the index assessment, defined as UACR ≥30 mg/g (28). In complementary analyses, UACR was analysed as a continuous outcome after log_10_ transformation.

### 2.5 Statistical analysis

Approximately normally distributed continuous variables are summarised as mean ± standard deviation (SD), whereas skewed variables are summarised as median with first and third quartiles. Categorical variables are reported as *n* (%). Between-group comparisons used the two-sample Student's t test, Welch's *t* test, Mann–Whitney U test, Pearson *χ*^2^ test, or Fisher's exact test, as appropriate. To assess potential selection associated with missing index components, available characteristics were compared between participants included in the final analysis and those excluded because of missing data required to calculate the IR-related indices, using absolute standardised mean differences (SMDs). An absolute SMD ≥0.20 was considered indicative of a potentially meaningful between-group imbalance (29). No imputation was performed because all 509 participants included in the final analysis had complete data for the variables used in the analyses.

Associations between each IR-related index and albuminuria were evaluated using separate logistic regression models. Each index was analysed both continuously (per 1-SD increment) and categorically using sample-specific quartiles (Supplementary Table S1), with the lowest quartile as the reference and no prespecified hierarchy between the two parameterisations. Overall associations across quartiles in logistic regression models were assessed using likelihood-ratio tests. The *P*-value for trend was calculated by modelling quartile categories as an ordinal variable. Covariates were selected based on clinical relevance and previous literature regarding factors associated with IR-related metabolic profiles or albuminuria. ACEI/ARB use was included in the primary adjustment set because of its established relationship with albuminuria and kidney outcomes (28). To assess potential residual confounding related to antihypertensive treatment, *β*-blocker, CCB, and diuretic use were additionally included in sensitivity analyses. Model 1 adjusted for age and sex. Model 2 additionally adjusted for current smoking, current alcohol use, ACEI/ARB use, and duration of hypertension. Model 3 further adjusted for Scr, UA, and HbA1c. Odds ratios (ORs) are reported with 95% confidence intervals (CIs).

Restricted cubic spline (RCS) models adjusted for the Model 3 covariates were used to examine the shape of the associations between the standardised indices and albuminuria. Four knots were set at the 5th, 35th, 65th, and 95th percentiles of each standardised index, with 0 SD as the reference (30). The overall association was evaluated by jointly testing all spline terms, and non-linearity was assessed by jointly testing the spline terms beyond the linear component.

Linear regression analyses examined the associations of the standardised and quartile-based IR-related indices with log_10_-transformed UACR using the same Model 1–3 adjustment sequence. Overall associations across quartiles in linear regression models were assessed using 3-degree-of-freedom partial *F* tests.

Two sensitivity analyses were performed for the logistic regression analyses. First, *β*-blocker, CCB, and diuretic use were additionally included in Model 3. Second, Model 3 was repeated after excluding participants with UACR ≥300 mg/g; for this analysis, quartile categories were defined using the same cut-off values as in the full cohort.

Given the uncertain causal role of MAP in this cross-sectional setting, MAP was examined using two distinct analytical approaches. In supplementary logistic regression models, MAP was additionally included in Model 3 to characterise the associations conditional on concurrent arterial pressure. Separately, exploratory indirect-effect analyses were conducted to assess whether the observed associations contained a statistical indirect component involving MAP (31–33). Each IR-related index was standardised to a z-score and evaluated per 1-SD increment. A multivariable linear regression model was fitted with MAP as the dependent variable, and a multivariable logistic regression model was fitted with albuminuria as the dependent variable. Both models included the Model 3 covariates. No interaction term between the index and MAP was included in the outcome model. Indirect, direct, and total effects were estimated on the absolute probability-difference scale, and the estimated proportion mediated was calculated as the indirect effect divided by the total effect. Percentile-based 95% CIs were derived from 5,000 non-parametric bootstrap resamples. These analyses were interpreted as exploratory statistical decomposition rather than evidence of temporal ordering or causal mediation.

Multicollinearity was assessed using variance inflation factors (VIFs), with a VIF of 5 used as the threshold for potentially concerning multicollinearity (34). Across Model 3, the medication-adjusted sensitivity models, and the MAP-adjusted supplementary models for the three IR-related indices, VIFs for all predictors ranged from 1.043 to 2.130; no meaningful multicollinearity was identified. All tests were two-sided, with *P* < 0.05 considered statistically significant. No formal adjustment for multiple comparisons was applied across the multiple related analyses; given their partly exploratory and complementary nature, interpretation emphasised effect estimates and 95% CIs rather than individual *P*-values alone (35). Analyses were performed using R version 4.5.2. RCS analyses were performed using the *rms* package, and indirect-effect analyses were performed using the *mediation* package.

## 3 Results

### 3.1 Study population and participant characteristics

The final analysis included 509 of the 792 patients screened (Figure 1). Supplementary Table S2 summarises the detailed causes of secondary hypertension, components of severe comorbidities, and, for the 66 participants excluded because of incomplete data for IR-index calculation, the specific variables that were missing. In an available-case comparison between participants included in the final analysis and those excluded because of missing data required to calculate the IR-related indices, absolute SMDs ≥0.20 were observed for current alcohol use, DBP, MAP, diuretic use, statin use, eGFR, and TC. The absolute SMDs for UACR and albuminuria prevalence were 0.130 and 0.060, respectively (Supplementary Table S3). Among the included participants, 99 (19.4%) had albuminuria at the index assessment. Of these, 87 (87.9%) had UACR values of 30–299 mg/g and 12 (12.1%) had UACR values ≥300 mg/g.

Characteristics according to albuminuria status are presented in Table 1. Compared with participants without albuminuria, those with albuminuria were younger and had higher BMI, SBP, DBP, and MAP. ACEI/ARB, *β*-blocker, CCB, and diuretic use were also more frequent among participants with albuminuria. Participants with albuminuria had higher Scr, UA, and TG concentrations, but lower HbA1c levels. TyG-BMI was higher in participants with albuminuria than in those without albuminuria [231.80 (213.41, 269.88) vs. 216.71 (198.77, 243.32); *P* < 0.001], as was METS-IR [42.72 (37.92, 49.10) vs. 40.22 (35.69, 44.56); *P* < 0.001]. The TyG index was also higher in the albuminuria group (8.74 ± 0.64 vs. 8.59 ± 0.54; *P* = 0.026).

**Table 1:** Characteristics of the participants by albuminuria status.

Characteristic	Overall (*n* = 509)	Without albuminuria (*n* = 410)	With albuminuria (*n* = 99)	*P*-value
Clinical characteristics				
Age, years	45.93 ± 13.82	47.13 ± 13.69	40.95 ± 13.31	<0.001
Male, *n* (%)	317 (62.3)	249 (60.7)	68 (68.7)	0.143
Current smoking, *n* (%)	150 (29.5)	121 (29.5)	29 (29.3)	0.966
Current alcohol use, *n* (%)	132 (25.9)	109 (26.6)	23 (23.2)	0.494
Duration of hypertension, years	2.00 (0.50–7.00)	2.00 (0.50–8.00)	2.00 (0.50–6.00)	0.652
History of CHD, *n* (%)	38 (7.5)	33 (8.0)	5 (5.1)	0.308
BMI, kg/m^2^	25.68 (23.44–28.08)	25.34 (23.31–27.68)	26.30 (24.20–29.89)	0.003
SBP, mmHg	145.00 (132.00–162.00)	142.00 (130.00–157.00)	163.00 (143.50–178.50)	<0.001
DBP, mmHg	95.00 (85.00–106.00)	93.00 (83.00–102.75)	105.00 (92.50–118.00)	<0.001
MAP, mmHg	111.33 (101.33–123.00)	109.33 (99.67–119.92)	122.33 (110.00–136.17)	<0.001
Medication use				
ACEI/ARB use, *n* (%)	372 (73.1)	289 (70.5)	83 (83.8)	0.007
*β*-blocker use, *n* (%)	145 (28.5)	106 (25.9)	39 (39.4)	0.007
CCB use, *n* (%)	357 (70.1)	276 (67.3)	81 (81.8)	0.005
Diuretic use, *n* (%)	101 (19.8)	67 (16.3)	34 (34.3)	<0.001
Statin use, *n* (%)	277 (54.4)	221 (53.9)	56 (56.6)	0.633
SGLT2i use, *n* (%)	3 (0.6)	1 (0.2)	2 (2.0)	0.098
Laboratory measurements				
FPG, mmol/L	4.97 ± 0.62	4.97 ± 0.63	4.96 ± 0.61	0.835
Hb, g/L	149.00 (135.00–161.00)	149.00 (134.00–161.00)	153.00 (138.00–162.50)	0.279
HbA1c, %	5.50 (5.20–5.70)	5.50 (5.30–5.78)	5.40 (5.10–5.60)	<0.001
UACR, mg/g	10.53 (5.54–22.66)	8.28 (4.73–14.36)	74.89 (43.88–146.41)	—
Scr, μmol/L	63.00 (51.00–74.00)	61.50 (50.00–72.00)	65.00 (56.50–79.00)	0.008
eGFR, mL/min/1.73 m^2^	111.36 (102.39–119.95)	111.45 (102.65–119.54)	111.35 (101.44–120.68)	0.748
UA, μmol/L	346.00 (282.00–429.00)	344.00 (278.00–426.25)	365.00 (309.00–470.00)	0.024
TG, mmol/L	1.37 (0.97–2.08)	1.35 (0.96–2.01)	1.56 (1.08–2.51)	0.012
TC, mmol/L	4.18 ± 0.91	4.15 ± 0.92	4.28 ± 0.89	0.210
LDL-C, mmol/L	2.60 ± 0.79	2.58 ± 0.79	2.67 ± 0.82	0.273
HDL-C, mmol/L	0.99 (0.86–1.15)	1.00 (0.86–1.15)	0.97 (0.83–1.14)	0.206
IR-related metabolic indices				
TyG index	8.62 ± 0.56	8.59 ± 0.54	8.74 ± 0.64	0.026
TyG-BMI	220.25 (200.58–247.55)	216.71 (198.77–243.32)	231.80 (213.41–269.88)	<0.001
METS-IR	40.47 (36.05–45.55)	40.22 (35.69–44.56)	42.72 (37.92–49.10)	<0.001

ACEI, angiotensin-converting enzyme inhibitor; ARB, angiotensin receptor blocker; BMI, body mass index; CCB, calcium channel blocker; CHD, coronary heart disease; DBP, diastolic blood pressure; eGFR, estimated glomerular filtration rate; FPG, fasting plasma glucose; Hb, haemoglobin; HbA1c, glycated haemoglobin A1c; HDL-C, high-density lipoprotein cholesterol; IR, insulin resistance; LDL-C, low-density lipoprotein cholesterol; MAP, mean arterial pressure; METS-IR, metabolic score for insulin resistance; SBP, systolic blood pressure; Scr, serum creatinine; SGLT2i, sodium-glucose cotransporter 2 inhibitor; TC, total cholesterol; TG, triglycerides; TyG, triglyceride-glucose; TyG-BMI, triglyceride-glucose body mass index; UA, serum uric acid; UACR, urinary albumin-to-creatinine ratio.

Data are presented as mean ± SD, median (first and third quartiles), or *n* (%). Albuminuria was defined as UACR ≥ 30 mg/g. All *P*-values are two-sided. Continuous variables were compared using Student's t test, Welch's *t* test, or the Mann–Whitney U test, as appropriate. Categorical variables were compared using the Pearson *χ*^2^ test or Fisher's exact test, as appropriate. No between-group *P*-value was calculated for UACR because albuminuria status was defined according to UACR.

### 3.2 Associations of IR-related metabolic indices with albuminuria

Albuminuria prevalence was highest in the fourth quartile of all three indices: 28.3% for TyG-BMI, 27.6% for METS-IR, and 26.0% for the TyG index (Figure 2, Supplementary Figure S1). In unadjusted logistic regression analyses, each 1-SD increment in TyG-BMI, METS-IR, and the TyG index was associated with higher odds of albuminuria. These associations remained after sequential adjustment. In Model 3, the ORs per 1-SD increment were 1.610 (95% CI, 1.262–2.055; *P* < 0.001) for TyG-BMI, 1.557 (95% CI, 1.215–1.995; *P* < 0.001) for METS-IR, and 1.436 (95% CI, 1.128–1.829; *P* = 0.003) for the TyG index. In the Model 3 quartile analyses, participants in the highest quartile had higher odds of albuminuria than those in the lowest quartile for TyG-BMI (OR, 2.464; 95% CI, 1.228–4.943; *P* = 0.011), METS-IR (OR, 2.665; 95% CI, 1.268–5.600; *P* = 0.010), and the TyG index (OR, 2.137; 95% CI, 1.092–4.181; *P* = 0.027). The overall test across quartiles was statistically significant for TyG-BMI (*P* = 0.018), but not for METS-IR (*P* = 0.059) or the TyG index (*P* = 0.099) (Table 2).

**Figure 2:**
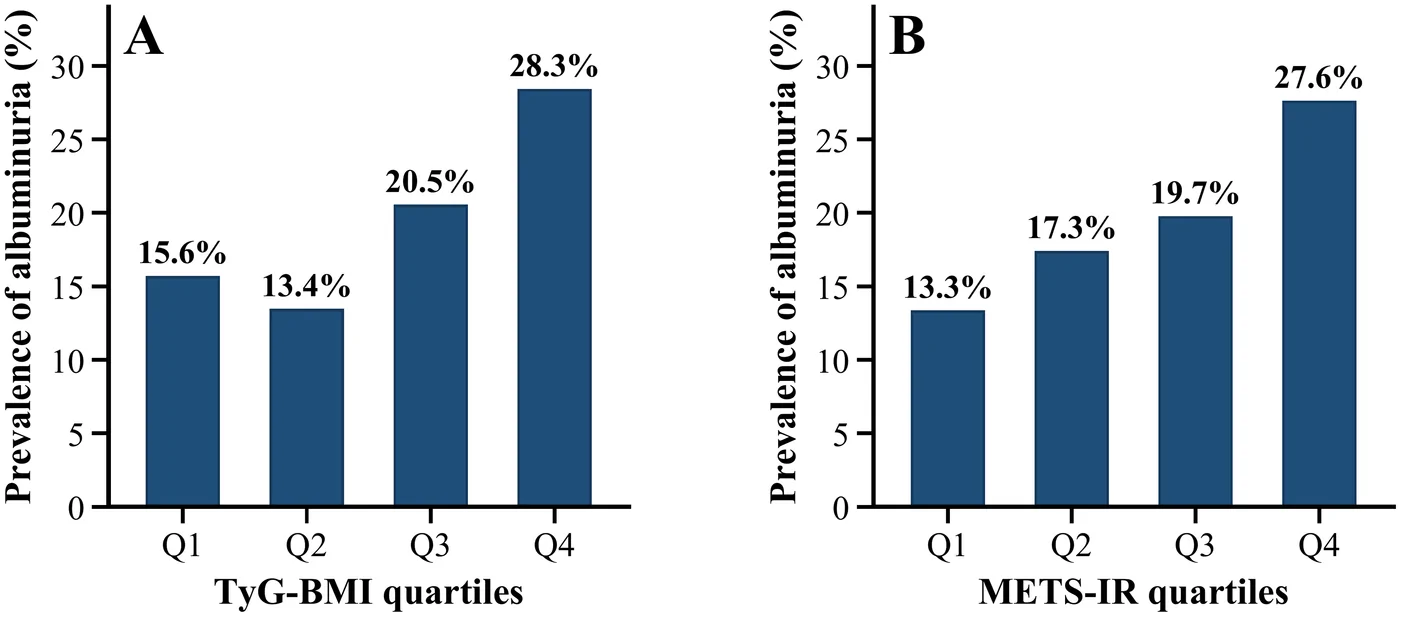
Prevalence of albuminuria across quartiles of TyG-BMI and METS-IR. The bars show the unadjusted prevalence of albuminuria across quartiles of TyG-BMI **(A)** and METS-IR **(B)**. Albuminuria was defined as UACR ≥30 mg/g. Q1–Q4 indicate the first to fourth quartiles, respectively. METS-IR, metabolic score for insulin resistance; TyG-BMI, triglyceride-glucose body mass index; UACR, urinary albumin-to-creatinine ratio.

**Table 2:** Associations of insulin resistance-related metabolic indices with albuminuria.

IR-related metabolic index	Analysis	Unadjusted		Model 1		Model 2		Model 3		Model 3 + MAP	
		OR (95% CI)	*P*-value	OR (95% CI)	*P*-value	OR (95% CI)	*P*-value	OR (95% CI)	*P*-value	OR (95% CI)	*P*-value
TyG-BMI	Per 1-SD increment	1.557 (1.255–1.932)	<0.001	1.485 (1.191–1.852)	<0.001	1.507 (1.200–1.892)	<0.001	1.610 (1.262–2.055)	<0.001	1.502 (1.161–1.941)	0.002
	Q1 (reference)	1.000 (reference)	—	1.000 (reference)	—	1.000 (reference)	—	1.000 (reference)	—	1.000 (reference)	—
	Q2	0.835 (0.415–1.679)	0.612	0.864 (0.421–1.772)	0.690	0.851 (0.412–1.758)	0.662	0.918 (0.438–1.923)	0.821	0.835 (0.386–1.806)	0.646
	Q3	1.390 (0.731–2.644)	0.315	1.357 (0.685–2.687)	0.381	1.321 (0.662–2.635)	0.430	1.538 (0.753–3.142)	0.237	1.246 (0.587–2.642)	0.567
	Q4	2.136 (1.157–3.946)	0.015	1.951 (1.023–3.718)	0.042	2.025 (1.049–3.907)	0.035	2.464 (1.228–4.943)	0.011	2.043 (0.990–4.219)	0.053
	*P* for overall	—	0.015	—	0.060	—	0.047	—	0.018	—	0.074
	*P* for trend	—	0.004	—	0.015	—	0.013	—	0.004	—	0.024
METS-IR	Per 1-SD increment	1.531 (1.234–1.899)	<0.001	1.447 (1.155–1.812)	0.001	1.470 (1.167–1.852)	0.001	1.557 (1.215–1.995)	<0.001	1.465 (1.128–1.903)	0.004
	Q1 (reference)	1.000 (reference)	—	1.000 (reference)	—	1.000 (reference)	—	1.000 (reference)	—	1.000 (reference)	—
	Q2	1.368 (0.688–2.719)	0.371	1.304 (0.640–2.658)	0.465	1.351 (0.658–2.774)	0.413	1.482 (0.711–3.089)	0.294	1.246 (0.574–2.703)	0.579
	Q3	1.600 (0.817–3.134)	0.170	1.584 (0.772–3.250)	0.210	1.677 (0.810–3.473)	0.164	1.937 (0.911–4.115)	0.086	1.760 (0.801–3.866)	0.159
	Q4	2.484 (1.307–4.720)	0.005	2.137 (1.072–4.261)	0.031	2.221 (1.105–4.465)	0.025	2.665 (1.268–5.600)	0.010	2.129 (0.979–4.630)	0.057
	*P* for overall	—	0.034	—	0.153	—	0.131	—	0.059	—	0.209
	*P* for trend	—	0.004	—	0.023	—	0.019	—	0.007	—	0.035
TyG index	Per 1-SD increment	1.326 (1.060–1.657)	0.013	1.345 (1.072–1.688)	0.011	1.381 (1.091–1.747)	0.007	1.436 (1.128–1.829)	0.003	1.349 (1.048–1.735)	0.020
	Q1 (reference)	1.000 (reference)	—	1.000 (reference)	—	1.000 (reference)	—	1.000 (reference)	—	1.000 (reference)	—
	Q2	0.952 (0.488–1.858)	0.886	0.981 (0.497–1.938)	0.956	0.993 (0.498–1.980)	0.985	1.084 (0.535–2.194)	0.824	0.963 (0.461–2.009)	0.919
	Q3	1.249 (0.658–2.369)	0.496	1.340 (0.696–2.583)	0.381	1.354 (0.695–2.637)	0.373	1.478 (0.746–2.931)	0.263	1.188 (0.576–2.451)	0.642
	Q4	1.789 (0.969–3.303)	0.063	1.824 (0.973–3.422)	0.061	1.939 (1.015–3.702)	0.045	2.137 (1.092–4.181)	0.027	1.786 (0.885–3.604)	0.106
	*P* for overall	—	0.159	—	0.165	—	0.126	—	0.099	—	0.270
	*P* for trend	—	0.038	—	0.035	—	0.026	—	0.016	—	0.078

ACEI, angiotensin-converting enzyme inhibitor; ARB, angiotensin receptor blocker; BMI, body mass index; CI, confidence interval; HbA1c, glycated haemoglobin A1c; IR, insulin resistance; MAP, mean arterial pressure; METS-IR, metabolic score for insulin resistance; OR, odds ratio; Scr, serum creatinine; SD, standard deviation; TyG, triglyceride-glucose; TyG-BMI, triglyceride-glucose body mass index; UA, serum uric acid; UACR, urinary albumin-to-creatinine ratio.

ORs and 95% CIs were estimated using binary logistic regression. For continuous analyses, ORs are reported per 1-SD increment in each index; quartile analyses use Q1 as the reference. *P* for overall was obtained using a likelihood-ratio test comparing nested models with and without the quartile indicator variables. *P* for trend was obtained by entering quartile categories as an ordinal variable. Albuminuria at the index assessment was defined as UACR ≥30 mg/g. All *P*-values are two-sided. Model 1 adjusted for age and sex. Model 2 additionally adjusted for current smoking, current alcohol use, ACEI/ARB use, and duration of hypertension. Model 3 further adjusted for Scr, UA, and HbA1c. The supplementary model (Model 3 + MAP) additionally adjusted for MAP. All analyses included 509 participants, of whom 99 had albuminuria at the index assessment.

In the RCS analyses, the overall spline tests were statistically significant for all three indices, with no statistical evidence of departure from linearity (Figure 3, Supplementary Figure S2).

**Figure 3:**
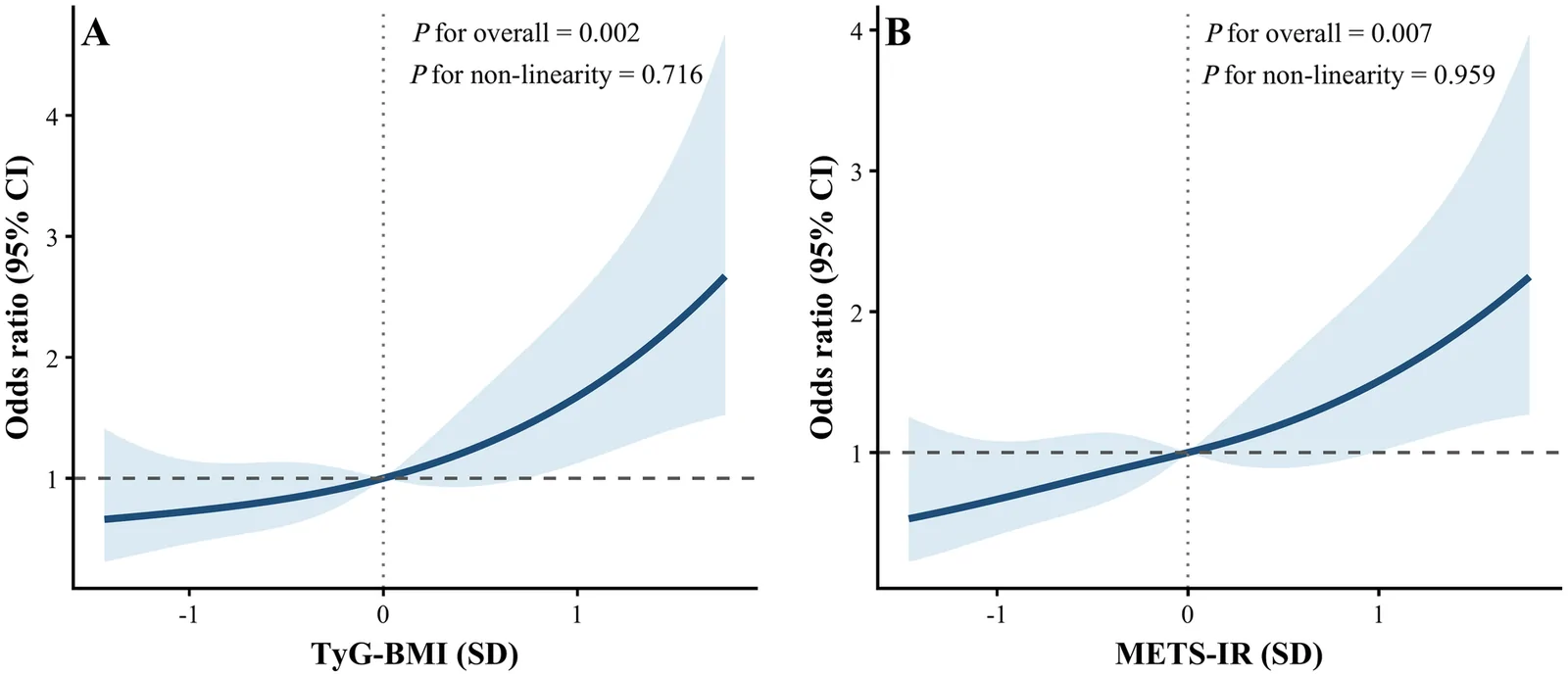
Restricted cubic spline associations of TyG-BMI and METS-IR with albuminuria. Restricted cubic spline analyses showing the adjusted associations of standardised TyG-BMI **(A)** and METS-IR **(B)** with albuminuria. Solid curves indicate odds ratios, and shaded areas indicate 95% confidence intervals. Horizontal dashed lines indicate an odds ratio of 1, and vertical dotted lines indicate the reference value of 0 SD. Models were adjusted for age, sex, current smoking, current alcohol use, ACEI/ARB use, duration of hypertension, serum creatinine, uric acid, and glycated haemoglobin A1c. *P*-values are shown for the overall association and non-linearity. CI, confidence interval; METS-IR, metabolic score for insulin resistance; OR, odds ratio; SD, standard deviation; TyG-BMI, triglyceride-glucose body mass index.

Complementary multivariable linear regression analyses yielded broadly consistent findings. All three indices were positively associated with log_10_-transformed UACR when modelled continuously, and the overall tests across quartiles were statistically significant for all three indices (Supplementary Table S4).

In the sensitivity analyses, after additional adjustment for *β*-blocker, CCB, and diuretic use, the continuous associations remained positive and statistically significant for all three IR-related indices, whereas none of the overall tests across quartiles reached statistical significance (Supplementary Table S5). After excluding participants with UACR ≥300 mg/g, the continuous associations remained positive and statistically significant for all three indices; the overall tests across quartiles were statistically significant for TyG-BMI and METS-IR, but not for the TyG index (Supplementary Table S6).

After additional adjustment for MAP, the continuous associations of all three IR-related indices with albuminuria were attenuated but remained statistically significant, with ORs per 1-SD increment of 1.502 (95% CI, 1.161–1.941) for TyG-BMI, 1.465 (95% CI, 1.128–1.903) for METS-IR, and 1.349 (95% CI, 1.048–1.735) for the TyG index. In the quartile analyses, neither the overall tests across quartiles nor the Q4-vs.-Q1 comparisons were statistically significant for any of the three indices (Table 2).

### 3.3 Exploratory indirect-effect analysis of mean arterial pressure

Exploratory indirect-effect analyses yielded statistically significant indirect-effect estimates involving MAP for TyG-BMI (0.0169; 95% CI, 0.0051–0.0304) and METS-IR (0.0160; 95% CI, 0.0041–0.0309), corresponding to estimated mediation proportions of 21.8% (95% CI, 7.0%–52.3%) and 22.1% (95% CI, 6.0%–56.8%), respectively. The indirect-effect estimate for the TyG index was not statistically significant (0.0093; 95% CI, −0.0006 to 0.0203) (Table 3).

**Table 3:** Exploratory indirect-effect analyses involving MAP in associations of IR-related metabolic indices with albuminuria.

IR-related metabolic index	Indirect effect		Direct effect		Total effect		Estimated proportion mediated	
	Estimate (95% CI)	*P*-value	Estimate (95% CI)	*P*-value	Estimate (95% CI)	*P*-value	Estimate (95% CI)	*P*-value
TyG-BMI	0.0169 (0.0051 to 0.0304)	0.006	0.0604 (0.0173 to 0.1088)	0.006	0.0773 (0.0319 to 0.1272)	<0.001	21.8% (7.0% to 52.3%)	0.006
METS-IR	0.0160 (0.0041 to 0.0309)	0.008	0.0567 (0.0137 to 0.1019)	0.007	0.0727 (0.0286 to 0.1199)	0.001	22.1% (6.0% to 56.8%)	0.010
TyG index	0.0093 (−0.0006 to 0.0203)	0.067	0.0433 (0.0026 to 0.0863)	0.036	0.0526 (0.0108 to 0.0965)	0.016	17.7% (−3.4% to 65.5%)	0.081

ACEI, angiotensin-converting enzyme inhibitor; ARB, angiotensin receptor blocker; CI, confidence interval; HbA1c, glycated haemoglobin A1c; IR, insulin resistance; MAP, mean arterial pressure; METS-IR, metabolic score for insulin resistance; Scr, serum creatinine; SD, standard deviation; TyG, triglyceride-glucose; TyG-BMI, triglyceride-glucose body mass index; UA, serum uric acid.

Effects were estimated on the absolute probability-difference scale for a 1-SD contrast in each standardised IR-related index, from the mean to 1 SD above the mean, using an exploratory counterfactual mediation framework. MAP was modelled as a continuous mediator. The mediator model used linear regression, whereas the albuminuria outcome model used logistic regression and did not include an interaction term between the index and MAP. Both models adjusted for age, sex, current smoking, current alcohol use, ACEI/ARB use, duration of hypertension, Scr, UA, and HbA1c. The 95% CIs were derived using the percentile method from 5,000 non-parametric bootstrap resamples. The estimated mediation proportion was calculated as the indirect effect divided by the total effect. All analyses included 509 participants. These estimates were interpreted as exploratory statistical decomposition rather than evidence of temporal ordering or causal mediation.

## 4 Discussion

In this cross-sectional study of patients with primary hypertension without diabetes, TyG-BMI, METS-IR, and the TyG index showed broadly concordant positive associations with albuminuria, although the strength of statistical evidence varied across parameterisations and analyses. The weaker statistical evidence in the quartile-based analyses may partly reflect reduced information and precision after categorisation, while also indicating some sensitivity to exposure parameterisation. The overall direction of association was broadly preserved in the complementary and sensitivity analyses, and the RCS analyses provided no statistical evidence that the associations deviated from linearity. Additional adjustment for MAP attenuated the observed associations, and exploratory indirect-effect analyses suggested a statistical component involving MAP for TyG-BMI and METS-IR but not for the TyG index. These findings suggest that readily available IR-related metabolic indices may help characterise metabolic profiles associated with albuminuria in patients with primary hypertension without diabetes, although their potential clinical relevance requires further prospective evaluation.

The observed associations were broadly consistent in direction and magnitude with previous epidemiological evidence. In a population-based study, the highest vs. lowest quartiles of METS-IR and the TyG index were associated with albuminuria, with adjusted ORs of approximately 2.2 and 1.8, respectively, comparable to the corresponding estimates in the present study (13). Direct quantitative comparisons should nevertheless be made cautiously because of differences in study populations, outcome definitions, exposure scaling, and covariate adjustment. Beyond this overall consistency, the three indices capture overlapping but not identical aspects of metabolic status; their concurrent evaluation therefore allowed assessment of whether similar associations were observed across indices with different component profiles. However, because these indices share several components and are mathematically related, their concordant associations should not be interpreted as evidence of distinct biological pathways or independent effects. Moreover, without formal head-to-head analyses of discrimination, prediction, or incremental model performance, the present findings should not be interpreted as demonstrating the superiority of any one index over another.

The attenuation of the associations after additional adjustment for MAP suggests statistical overlap with concurrent haemodynamic burden and warrants cautious interpretation. The metabolic characteristics reflected by these indices have been linked to processes that may increase arterial pressure, including sympathetic and renin–angiotensin–aldosterone system activation, altered sodium handling, impaired vasodilation, and increased arterial stiffness (20–23). In turn, elevated systemic pressure may increase renal microvascular and glomerular haemodynamic stress, particularly when renal autoregulatory capacity is impaired, thereby increasing urinary albumin excretion (1, 19). Importantly, MAP reflects systemic arterial pressure rather than intraglomerular pressure and therefore cannot directly characterise renal microvascular haemodynamic burden (19, 24). Bidirectional relationships are also plausible, because abnormalities in renal sodium handling and pressure natriuresis may promote sodium retention and neurohormonal activation, thereby contributing to sustained blood pressure elevation (20–22). Thus, attenuation after MAP adjustment may reflect an intermediate haemodynamic component, confounding related to hypertension severity, or shared underlying disease burden. Because the metabolic indices, MAP, and UACR were assessed concurrently, their temporal sequence cannot be determined, and the indirect-effect estimates should not be interpreted as evidence of a causal MAP-mediated pathway (31, 33).

The metabolic profiles reflected by these indices may also be related to albuminuria through biological processes that are not fully captured by systemic arterial pressure. Adiposity, dyslipidaemia, and glucose-related metabolic abnormalities have been associated with endothelial dysfunction, oxidative stress and inflammation (7, 23, 36), and alterations in glomerular endothelial and podocyte function, potentially compromising the glomerular filtration barrier and increasing urinary albumin excretion (37–41). These mechanisms may provide biological plausibility for the observed associations but were not directly assessed in the present study.

Several baseline characteristics also warrant cautious interpretation. The participants with albuminuria were younger despite having higher blood pressure levels and more frequent antihypertensive medication use. This pattern may partly reflect hospital-based selection rather than a direct relationship between younger age and albuminuria. More frequent antihypertensive medication use may similarly reflect medication indications and treatment intensity rather than hypertension severity alone. The slightly lower HbA1c level among the participants with albuminuria should be interpreted in the context of the restricted glycaemic range resulting from the exclusion of patients with diabetes and the small absolute between-group difference.

This study has several limitations. First, the retrospective cross-sectional design precludes determination of temporal order and causal inference, and reverse causation cannot be excluded. The relatively wide confidence intervals around the estimated mediation proportions also indicate limited precision. Second, albuminuria was defined using a single UACR measurement and may therefore be affected by biological variability or transient misclassification rather than representing persistent albuminuria (28). Third, TyG-BMI, METS-IR, and the TyG index are surrogate metabolic indices and were not compared with a gold-standard measure of IR, such as the hyperinsulinaemic–euglycaemic clamp; therefore, the observed associations cannot be attributed specifically to IR itself. Fourth, selection bias related to UACR testing and data completeness remains possible. Although available characteristics were compared between included participants and those excluded because of missing data required to calculate the IR-related indices, imbalances in several characteristics were observed, and the single-centre hospitalised cardiology setting may limit generalisability. Fifth, some clinical classifications and measurements relied on routine clinical care rather than study-specific standardised procedures. No uniform screening protocol for secondary hypertension was applied, and some participants with unrecognised secondary hypertension may therefore have been misclassified as having primary hypertension. Similarly, blood pressure measurements were obtained from routine medical records rather than according to a study-specific standardised protocol or by ambulatory monitoring, which may have introduced measurement error, particularly in analyses involving MAP. Sixth, residual confounding related to hypertension severity, medication indications and treatment intensity, treatment adherence, and other unmeasured factors cannot be excluded. Finally, multiple related analyses were performed without formal multiplicity adjustment, which may increase the possibility of chance findings (35).

## 5 Conclusion

In conclusion, higher TyG-BMI, METS-IR, and TyG index values were associated with greater odds of albuminuria and higher UACR in patients with primary hypertension without diabetes, although the strength of the statistical evidence varied across exposure parameterisations. Exploratory analyses suggested a statistical indirect component involving MAP for TyG-BMI and METS-IR, but the cross-sectional design precludes inference regarding temporal ordering or causal mediation. Prospective studies with repeated UACR measurements are needed to confirm these associations, clarify their temporal relationships, and determine their clinical relevance.

## Data Availability

The datasets generated and/or analysed during the current study are not publicly available because they contain potentially sensitive clinical information and are subject to patient privacy and institutional restrictions. De-identified data may be available from the corresponding author upon reasonable request and subject to approval by the relevant ethics committee and institution.
