# Supplementary material for: Associations of insulin resistance-related metabolic indices with albuminuria in primary hypertension without diabetes: a retrospective cross-sectional study
Source: Front Med (Lausanne). 2026 Oct 7;13:1959589. doi: 10.3389/fmed.2026.1959589 (PMC13683226; doi:10.3389/fmed.2026.1959589)
Supplement: Supplementary file 1 [file Supplementaryfile1.docx]

# Supplementary Tables and Figures

**Supplementary Table S1.** Observed ranges and numbers of participants across quartiles of the insulin resistance-related metabolic indices

| **IR-related metabolic indices** | **Quartile** | **Observed range** | **n** |
| --- | --- | --- | --- |
| TyG-BMI | Q1 | 135.34–200.58 | 128 |
|  | Q2 | 200.84–220.25 | 127 |
|  | Q3 | 220.26–247.55 | 127 |
|  | Q4 | 248.11–399.62 | 127 |
| METS-IR | Q1 | 21.90–36.05 | 128 |
|  | Q2 | 36.08–40.47 | 127 |
|  | Q3 | 40.49–45.55 | 127 |
|  | Q4 | 45.60–73.73 | 127 |
| TyG index | Q1 | 7.014–8.216 | 128 |
|  | Q2 | 8.219–8.595 | 127 |
|  | Q3 | 8.598–9.024 | 127 |
|  | Q4 | 9.028–10.376 | 127 |

Quartiles were defined separately for each index based on its distribution in the final analytical sample. Ranges represent the observed minimum and maximum values within each quartile.

Abbreviations: IR, insulin resistance; METS-IR, metabolic score for insulin resistance; TyG, triglyceride-glucose; TyG-BMI, triglyceride-glucose body mass index.

**Supplementary Table S2.** Detailed causes of secondary hypertension, types of severe comorbidities, and missing components among excluded participants

| **Exclusion category** | **Specific reason or missing component** | **n** |
| --- | --- | --- |
| **Secondary hypertension** | Renal parenchymal hypertension | 12 |
|  | Renal artery stenosis | 11 |
|  | Primary aldosteronism | 6 |
|  | Pheochromocytoma | 5 |
|  | Cushing syndrome | 3 |
|  | **Subtotal** | **37** |
| **Severe comorbidities** | Advanced malignancy | 3 |
|  | Severe hepatic dysfunction | 6 |
|  | Acute heart failure | 22 |
|  | **Subtotal** | **31** |
| **Missing components required to calculate the IR-related metabolic indices** | BMI | 53 |
|  | FPG | 5 |
|  | TG | 11 |
|  | HDL-C | 17 |
|  | **Participants excluded because of ≥1 missing component** | **66** |

Participants excluded because of missing components could have more than one missing variable; therefore, the counts for BMI, FPG, TG, and HDL-C are not mutually exclusive and do not sum to 66. Severe hepatic dysfunction was defined as aminotransferase concentrations >3 times the upper limit of normal.

Abbreviations: BMI, body mass index; FPG, fasting plasma glucose; HDL-C, high-density lipoprotein cholesterol; IR, insulin resistance; TG, triglycerides.

**Supplementary Table S3.** Characteristics of included participants and those excluded because of missing components required to calculate the insulin resistance-related metabolic indices

| **Characteristic** | **Included participants (n = 509)** | **Excluded participants (n = 66)** | **Available n** | **Absolute SMD** | ***P* value** |
| --- | --- | --- | --- | --- | --- |
| **Clinical characteristics** |  |  |  |  |  |
| Age, years | 45.93 ± 13.82 | 48.42 ± 15.07 | 65 | 0.172 | 0.210 |
| Male, n (%) | 317 (62.3) | 41 (63.1) | 65 | 0.016 | 0.900 |
| Current smoking, n (%) | 150 (29.5) | 16 (25.0) | 64 | 0.101 | 0.458 |
| Current alcohol use, n (%) | 132 (25.9) | 11 (17.2) | 64 | 0.214 | 0.128 |
| Duration of hypertension, years | 2.00 (0.50–7.00) | 3.00 (1.00–10.00) | 64 | 0.123 | 0.119 |
| History of CHD, n (%) | 38 (7.5) | 2 (3.1) | 64 | 0.195 | 0.296 |
| SBP, mmHg | 145.00 (132.00–162.00) | 140.50 (130.00–157.75) | 62 | 0.151 | 0.284 |
| DBP, mmHg | 95.00 (85.00–106.00) | 90.00 (77.00–104.00) | 62 | 0.280 | 0.043 |
| MAP, mmHg | 111.33 (101.33–123.00) | 104.83 (95.75–123.92) | 62 | 0.243 | 0.083 |
| **Medication use** |  |  |  |  |  |
| β-blocker use, n (%) | 145 (28.5) | 20 (31.7) | 63 | 0.071 | 0.590 |
| CCB use, n (%) | 357 (70.1) | 45 (70.3) | 64 | 0.004 | 0.977 |
| Diuretic use, n (%) | 101 (19.8) | 6 (9.4) | 64 | 0.300 | 0.043 |
| Statin use, n (%) | 277 (54.4) | 44 (68.8) | 64 | 0.298 | 0.029 |
| SGLT2i use, n (%) | 3 (0.6) | 0 (0.0) | 64 | 0.109 | 1.000 |
| **Laboratory measurements** |  |  |  |  |  |
| HbA1c, % | 5.50 (5.20–5.70) | 5.60 (5.30–5.80) | 61 | 0.133 | 0.266 |
| UACR, mg/g | 10.53 (5.54–22.66) | 12.74 (7.78–21.15) | 64 | 0.130 | 0.126 |
| Albuminuria, n (%) | 99 (19.4) | 14 (21.9) | 64 | 0.060 | 0.646 |
| Scr, μmol/L | 63.00 (51.00–74.00) | 65.00 (55.00–75.00) | 61 | 0.154 | 0.223 |
| eGFR, mL/min/1.73 m² | 111.36 (102.39–119.95) | 108.05 (98.92–118.29) | 61 | 0.248 | 0.095 |
| UA, μmol/L | 346.00 (282.00–429.00) | 352.00 (287.00–410.00) | 61 | 0.025 | 0.837 |
| TC, mmol/L | 4.18 ± 0.91 | 4.41 ± 0.97 | 50 | 0.252 | 0.103 |
| LDL-C, mmol/L | 2.60 ± 0.79 | 2.74 ± 0.89 | 49 | 0.176 | 0.264 |

Data are presented as mean ± standard deviation (SD), median (first and third quartiles), or n (%). Available n denotes the number of non-missing observations among excluded participants for each variable. Absolute standardised mean differences (SMDs) were calculated using pooled standard deviations for continuous variables and pooled proportions for binary variables; UACR was log10-transformed for SMD calculation. An absolute SMD ≥0.20 was considered potentially meaningful. P values were derived using Welch’s t test, Mann–Whitney U test, Pearson χ² test, or Fisher's exact test, as appropriate. Albuminuria was defined as UACR ≥30 mg/g.

Abbreviations: CCB, calcium channel blocker; CHD, coronary heart disease; DBP, diastolic blood pressure; eGFR, estimated glomerular filtration rate; HbA1c, glycated haemoglobin A1c; LDL-C, low-density lipoprotein cholesterol; MAP, mean arterial pressure; SBP, systolic blood pressure; Scr, serum creatinine; SGLT2i, sodium-glucose cotransporter 2 inhibitor; SMD, standardised mean difference; TC, total cholesterol; UA, serum uric acid; UACR, urinary albumin-to-creatinine ratio.

**Supplementary Table S4.** Associations of insulin resistance-related metabolic indices with log10-transformed urinary albumin-to-creatinine ratio

| **IR-related metabolic indices** | **Analysis** | **Unadjusted** | | **Model 1** | | **Model 2** | | **Model 3** | |
| --- | --- | --- | --- | --- | --- | --- | --- | --- | --- |
|  |  | ***β* (95% CI)** | ***P* value** | ***β* (95% CI)** | ***P* value** | ***β* (95% CI)** | ***P* value** | ***β* (95% CI)** | ***P* value** |
| **TyG-BMI** | Per 1-SD increment | 0.090 (0.044–0.136) | < 0.001 | 0.093 (0.046–0.140) | < 0.001 | 0.097 (0.050–0.144) | < 0.001 | 0.109 (0.061–0.156) | < 0.001 |
|  | Q1 (reference) | Reference | — | Reference | — | Reference | — | Reference | — |
|  | Q2 | −0.119 (−0.250–0.011) | 0.073 | −0.098 (−0.229–0.033) | 0.142 | −0.094 (−0.223–0.035) | 0.154 | −0.076 (−0.203–0.051) | 0.240 |
|  | Q3 | −0.024 (−0.154–0.106) | 0.718 | 0.000 (−0.134–0.135) | 0.995 | −0.005 (−0.137–0.128) | 0.947 | 0.027 (−0.104–0.158) | 0.685 |
|  | Q4 | 0.156 (0.025–0.286) | 0.020 | 0.161 (0.027–0.294) | 0.019 | 0.172 (0.040–0.304) | 0.011 | 0.205 (0.071–0.339) | 0.003 |
|  | *P* for overall | — | < 0.001 | — | 0.002 | — | < 0.001 | — | < 0.001 |
|  | *P* for trend | — | 0.008 | — | 0.006 | — | 0.004 | — | < 0.001 |
| **METS-IR** | Per 1-SD increment | 0.082 (0.036–0.129) | < 0.001 | 0.089 (0.040–0.137) | < 0.001 | 0.092 (0.044–0.141) | < 0.001 | 0.101 (0.051–0.150) | < 0.001 |
|  | Q1 (reference) | Reference | — | Reference | — | Reference | — | Reference | — |
|  | Q2 | 0.001 (−0.130–0.132) | 0.987 | 0.012 (−0.121–0.145) | 0.858 | 0.030 (−0.101–0.162) | 0.653 | 0.046 (−0.083–0.176) | 0.486 |
|  | Q3 | −0.041 (−0.173–0.090) | 0.536 | −0.011 (−0.149–0.126) | 0.870 | 0.004 (−0.132–0.139) | 0.956 | 0.024 (−0.110–0.159) | 0.723 |
|  | Q4 | 0.168 (0.037–0.299) | 0.012 | 0.171 (0.032–0.309) | 0.016 | 0.185 (0.048–0.322) | 0.009 | 0.209 (0.069–0.348) | 0.003 |
|  | *P* for overall | — | 0.010 | — | 0.024 | — | 0.019 | — | 0.010 |
|  | *P* for trend | — | 0.031 | — | 0.025 | — | 0.016 | — | 0.007 |
| **TyG index** | Per 1-SD increment | 0.051 (0.005–0.098) | 0.031 | 0.058 (0.011–0.104) | 0.015 | 0.063 (0.017–0.110) | 0.007 | 0.069 (0.023–0.115) | 0.004 |
|  | Q1 (reference) | Reference | — | Reference | — | Reference | — | Reference | — |
|  | Q2 | 0.049 (−0.083–0.181) | 0.469 | 0.057 (−0.073–0.187) | 0.391 | 0.063 (−0.065–0.191) | 0.338 | 0.084 (−0.043–0.210) | 0.196 |
|  | Q3 | 0.145 (0.013–0.277) | 0.032 | 0.160 (0.030–0.291) | 0.016 | 0.169 (0.040–0.298) | 0.010 | 0.190 (0.063–0.317) | 0.003 |
|  | Q4 | 0.112 (−0.020–0.244) | 0.096 | 0.119 (−0.012–0.249) | 0.075 | 0.134 (0.004–0.264) | 0.044 | 0.144 (0.015–0.274) | 0.030 |
|  | *P* for overall | — | 0.136 | — | 0.084 | — | 0.051 | — | 0.024 |
|  | *P* for trend | — | 0.042 | — | 0.029 | — | 0.015 | — | 0.010 |

*β* coefficients represent differences in mean log10-transformed UACR estimated using linear regression. For continuous analyses*, β* coefficients are reported per 1-SD increment in each index; quartile analyses use Q1 as the reference group. *P* for overall was obtained using a 3-degree-of-freedom partial F test for the quartile indicator variables. *P* for trend was obtained by modelling quartile categories as an ordinal variable. All *P* values are two-sided. Model 1 adjusted for age and sex. Model 2 additionally adjusted for current smoking, current alcohol use, ACEI/ARB use, and duration of hypertension. Model 3 further adjusted for Scr, UA, and HbA1c. All analyses included 509 participants.

Abbreviations: ACEI, angiotensin-converting enzyme inhibitor; ARB, angiotensin receptor blocker; CI, confidence interval; HbA1c, glycated haemoglobin A1c; IR, insulin resistance; METS-IR, metabolic score for insulin resistance; Scr, serum creatinine; SD, standard deviation; TyG, triglyceride-glucose; TyG-BMI, triglyceride-glucose body mass index; UA, serum uric acid; UACR, urinary albumin-to-creatinine ratio.

**Supplementary Table S5.** Sensitivity analysis of associations of insulin resistance-related metabolic indices with albuminuria after additional adjustment for β-blocker, calcium channel blocker, and diuretic use

| **Analysis** | **TyG-BMI** | | **METS-IR** | | **TyG index** | |
| --- | --- | --- | --- | --- | --- | --- |
|  | **OR (95% CI)** | ***P* value** | **OR (95% CI)** | ***P* value** | **OR (95% CI)** | ***P* value** |
| Per 1-SD increment | 1.460 (1.136–1.876) | 0.003 | 1.428 (1.107–1.843) | 0.006 | 1.383 (1.076–1.778) | 0.011 |
| Q1 (reference) | 1.000 (reference) | — | 1.000 (reference) | — | 1.000 (reference) | — |
| Q2 | 0.771 (0.361–1.650) | 0.503 | 1.257 (0.590–2.676) | 0.554 | 0.999 (0.483–2.069) | 0.999 |
| Q3 | 1.224 (0.583–2.570) | 0.593 | 1.650 (0.764–3.561) | 0.202 | 1.240 (0.612–2.513) | 0.550 |
| Q4 | 1.862 (0.909–3.815) | 0.089 | 2.028 (0.946–4.348) | 0.069 | 1.865 (0.931–3.734) | 0.079 |
| *P* for overall | — | 0.089 | — | 0.272 | — | 0.229 |
| *P* for trend | — | 0.036 | — | 0.050 | — | 0.058 |

Odds ratios (ORs) and 95% confidence intervals (CIs) were estimated using binary logistic regression. For continuous analyses, ORs are reported per 1-SD increment in each index; quartile analyses use Q1 as the reference. All models were adjusted for age, sex, current smoking, current alcohol use, ACEI/ARB use, duration of hypertension, Scr, UA, HbA1c, β-blocker use, CCB use, and diuretic use. P for overall was obtained using a likelihood-ratio test comparing nested models with and without the quartile indicator variables. P for trend was obtained by modelling quartile categories as an ordinal variable. Albuminuria was defined as UACR ≥30 mg/g. All analyses included 509 participants, of whom 99 had albuminuria.

Abbreviations: ACEI, angiotensin-converting enzyme inhibitor; ARB, angiotensin receptor blocker; CCB, calcium channel blocker; CI, confidence interval; HbA1c, glycated haemoglobin A1c; METS-IR, metabolic score for insulin resistance; OR, odds ratio; Scr, serum creatinine; SD, standard deviation; TyG, triglyceride-glucose; TyG-BMI, triglyceride-glucose body mass index; UA, serum uric acid; UACR, urinary albumin-to-creatinine ratio.

**Supplementary Table S6.** Sensitivity analysis of associations of insulin resistance-related metabolic indices with albuminuria after excluding participants with UACR ≥300 mg/g

| **Analysis** | **TyG-BMI** | | **METS-IR** | | **TyG index** | |
| --- | --- | --- | --- | --- | --- | --- |
|  | **OR (95% CI)** | ***P* value** | **OR (95% CI)** | ***P* value** | **OR (95% CI)** | ***P* value** |
| Per 1-SD increment | 1.744 (1.340–2.269) | < 0.001 | 1.656 (1.271–2.158) | < 0.001 | 1.499 (1.156–1.942) | 0.002 |
| Q1 (reference) | 1.000 (reference) | — | 1.000 (reference) | — | 1.000 (reference) | — |
| Q2 | 1.064 (0.480–2.357) | 0.879 | 1.369 (0.626–2.993) | 0.431 | 1.109 (0.522–2.354) | 0.788 |
| Q3 | 1.938 (0.903–4.156) | 0.089 | 2.010 (0.909–4.446) | 0.085 | 1.571 (0.762–3.239) | 0.221 |
| Q4 | 3.067 (1.441–6.529) | 0.004 | 3.009 (1.370–6.609) | 0.006 | 2.396 (1.181–4.862) | 0.015 |
| *P* for overall | — | 0.007 | — | 0.029 | — | 0.057 |
| *P* for trend | — | 0.001 | — | 0.003 | — | 0.008 |

Odds ratios (ORs) and 95% confidence intervals (CIs) were estimated using binary logistic regression. This sensitivity analysis excluded 12 participants with UACR ≥300 mg/g, leaving 497 participants for analysis. For continuous analyses, ORs are reported per 1-SD increment in each index; quartile analyses use Q1 as the reference. Quartile cutoffs derived from the full analytical cohort were retained in this sensitivity analysis. All models were adjusted for age, sex, current smoking, current alcohol use, ACEI/ARB use, duration of hypertension, Scr, UA, and HbA1c. P for overall was obtained using a likelihood-ratio test comparing nested models with and without the quartile indicator variables. P for trend was obtained by modelling quartile categories as an ordinal variable. In this restricted sample, albuminuria was defined as UACR 30–299 mg/g.

Abbreviations: ACEI, angiotensin-converting enzyme inhibitor; ARB, angiotensin receptor blocker; CI, confidence interval; HbA1c, glycated haemoglobin A1c; METS-IR, metabolic score for insulin resistance; OR, odds ratio; Scr, serum creatinine; SD, standard deviation; TyG, triglyceride-glucose; TyG-BMI, triglyceride-glucose body mass index; UA, serum uric acid; UACR, urinary albumin-to-creatinine ratio.

**Supplementary Figure S1. Prevalence of albuminuria across quartiles of the TyG index**

The bars show the unadjusted prevalence of albuminuria across quartiles of the TyG index. Albuminuria was defined as UACR ≥30 mg/g. Q1–Q4 indicate the first to fourth quartiles, respectively. TyG, triglyceride-glucose; UACR, urinary albumin-to-creatinine ratio.

**Supplementary Figure S2. Restricted cubic spline association of the TyG index with albuminuria**

Restricted cubic spline analysis showing the adjusted association of the standardised TyG index with albuminuria. The solid curve represents the odds ratio, and the shaded area represents the 95% confidence interval. The horizontal dashed line indicates an odds ratio of 1, and the vertical dotted line indicates the reference value of 0 SD. Four knots were placed at the 5th, 35th, 65th, and 95th percentiles of the standardised TyG index. The model was adjusted for age, sex, current smoking, current alcohol use, ACEI/ARB use, duration of hypertension, serum creatinine, serum uric acid, and glycated haemoglobin A1c. *P* values are shown for the overall association and non-linearity. ACEI, angiotensin-converting enzyme inhibitor; ARB, angiotensin receptor blocker; CI, confidence interval; OR, odds ratio; SD, standard deviation; TyG, triglyceride-glucose.
